# Pneumocephalus after the Treatment of an Inoperable Superior Sulcus Tumor with Chemoradiation

**DOI:** 10.1155/2017/3016517

**Published:** 2017-11-26

**Authors:** Ashley Albert, Robert Allbright, Todd Nichols, Edward Farley, Srinivasan Vijayakumar

**Affiliations:** ^1^Department of Radiation Oncology, University of Mississippi Medical Center, Jackson, MS, USA; ^2^Department of Radiology, University of Mississippi Medical Center, Jackson, MS, USA

## Abstract

**Background:**

Pneumocephalus is a rare phenomenon that can occur as a complication after operations involving the thoracic discs, following thoracotomy for tumor resection, and after an intracranial operation or cranial trauma. This complication frequently occurs when a tumor is located in the costovertebral angle and an operative intervention creates a tear in the dura resulting in a pleural-dural fistula.

**Case Presentation:**

We describe the case of a 58-year-old man with an inoperable superior sulcus tumor who developed pneumocephalus after the initiation of chemoradiation secondary to a pleural-dural fistula.

**Conclusions:**

Although a rare occurrence, pneumocephalus should be considered when patients with tumors in the superior sulcus treated with radiation develop neurologic symptoms characteristic of increased intracranial pressure.

## 1. Background

Pneumocephalus is an uncommon phenomenon, and most cases occur after resection of lung tumors as a result of the formation of an iatrogenic subarachnoid pleural fistula (ISPF). This type of fistula occurs when there is a disruption of both the dura and the parietal pleura which allows intradural air to accumulate [1]. Symptoms of pneumocephalus include headaches, altered mental status, focal deficits, seizures, and stroke. A characteristic finding of pneumocephalus is a subjective “splashing” sound induced by a rapid change in head position which is referred to as *bruit hydro aerique* [2].

The spinal nerves are formed by the dorsal and ventral roots, and these roots exit the dura through separate fenestrations [1]. Dural defects affecting these nerve roots can occur during a thoracotomy in which there is traction on a tumor adherent to the nerve and the nerve root is sacrificed intentionally or due to forceful rib retraction. Such disruptions can lead to the formation of an ISPF. During the respiratory cycle, inspiration results in negative intrathoracic pressure, and CSF is drawn into the pleural space. With expiration, positive intrathoracic pressure drives air in the subarachnoid space resulting in pneumocephalus. The combination of these pressure changes, and a valve-like mechanism can lead to a tension pneumocephalus which can be life threatening [3]. Pneumocephalus as a sequela of an ISPF usually leads to air with the ventricles and basilar cisterns, whereas head trauma or craniotomy may result in air located over the cerebral convexities [4].

Approximately half of the reported cases of pneumocephalus as a result of a pleural-dural fistula after a thoracotomy received neoadjuvant radiotherapy [5]. Pneumocephalus after radiation alone is rare and has been described in cases of definitive radiation for treatment of nasopharyngeal carcinoma. The authors of one such case report proposed that radiation resulted in the destruction of a tumor invading the base of the skull that then created a defect through which air entered the intracranial cavity resulting in pneumocephalus [6].

## 2. Case Presentation

**Figure 1:**
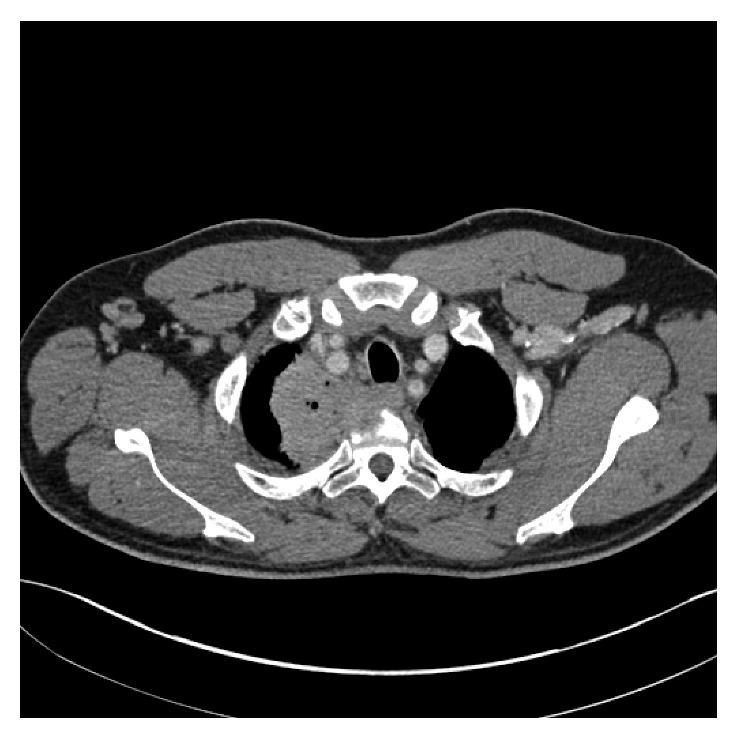
Computed tomography scan of the chest showing right superior sulcus mass invading a vertebral body.

A 58-year-old man initially presented with a 3-month history of cough, hemoptysis, right-sided chest and back pain, and progressive right-sided arm numbness and right-sided face weakness. A computed tomography (CT) scan revealed a large lung mass in the right superior sulcus with invasion of the C7 through T2 vertebral bodies, encasement of the right subclavian artery, compression of the right brachiocephalic vein, and possible invasion of the upper esophagus (Figure 1). Enlarged mediastinal, right hilar, and right supraclavicular lymph nodes concerning metastatic lymphadenopathy were also present. Magnetic resonance imaging (MRI) of the brain was negative for metastases. Bronchoscopy with a transbronchial biopsy was performed, and pathology was consistent with squamous cell carcinoma. The tumor was deemed inoperable due to extensive local invasion and lymph node involvement. Concurrent chemoradiation with carboplatin and Taxol was initiated. Given the concern for possible spinal cord compression, conformal radiation therapy was started emergently using AP/PA technique planned to a dose of 40 gray (Gy).

**Figure 2:**
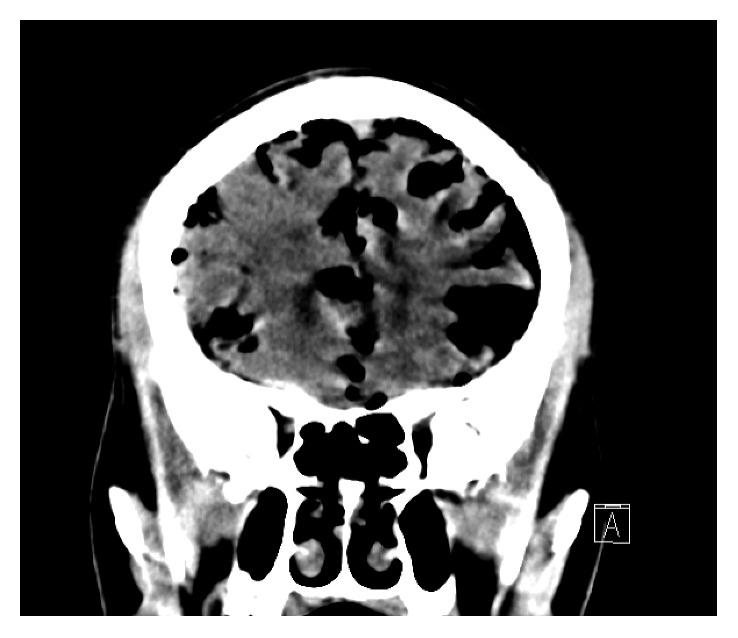
CT head with extensive pneumocephalus within the sulci and cisterns.

**Figure 3:**
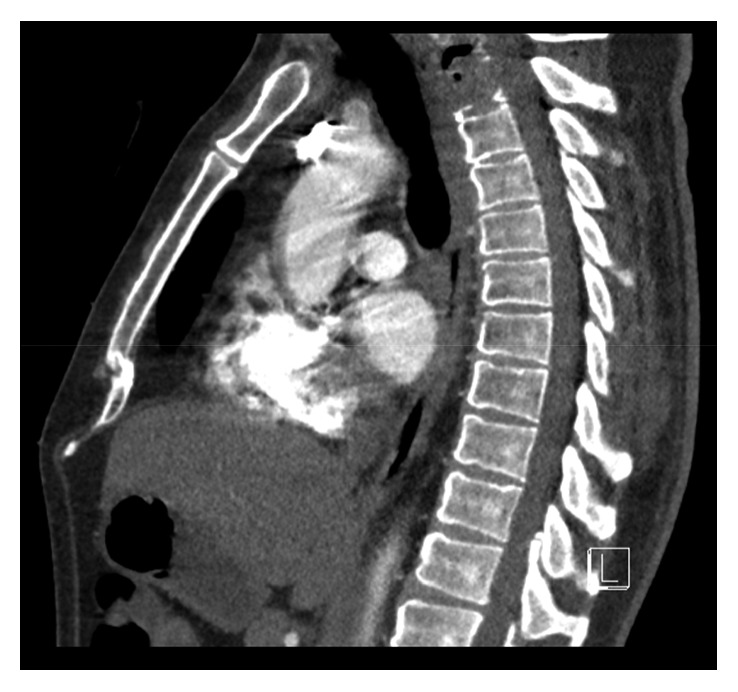
CT spine demonstrating local invasion of the tumor and air within the vertebral bodies.

**Figure 4:**
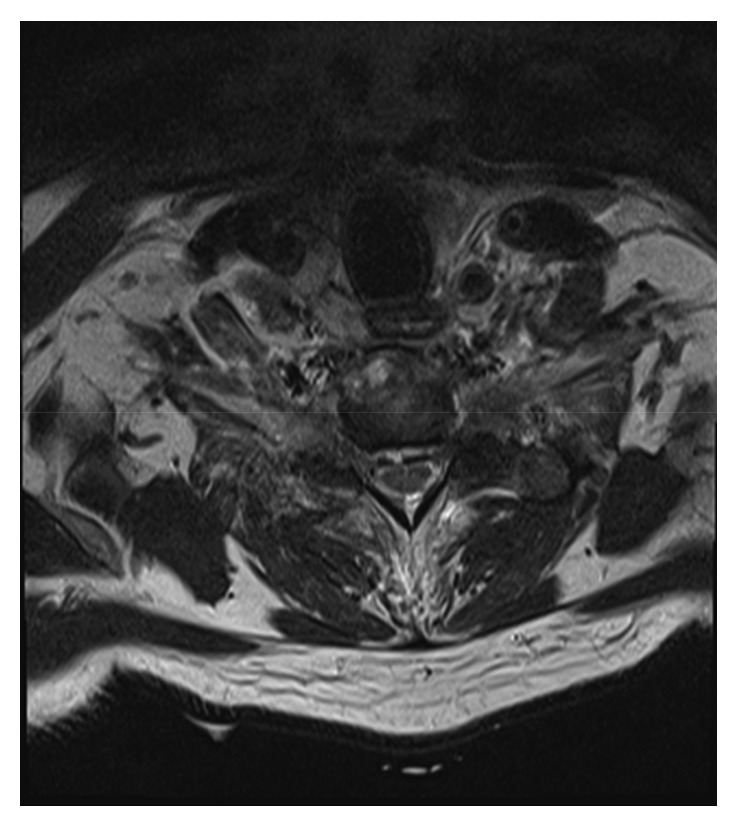
MRI thoracic spine showing local invasion of tumor.

After 32 Gy and 2 cycles of carboplatin and paclitaxel, the patient reported a severe headache that had been present for two days. CT scan of the head demonstrated interval development of extensive pneumocephalus tracking along the sulci and within the cisterns with mass effect on the frontal lobes (Figure 2). Foci of gas were also noted within the lower spinal canal. CT and MRI of the spine demonstrated air within the T1 vertebral body extending into the right facet of T1 and T2 (Figures 3 and 4). There was also a minimal amount of air in the central canal at the C5 and C6 vertebral bodies.

He was admitted due to these findings and followed by neurosurgery. Radiation and chemotherapy were discontinued. He was kept in a recumbent position while admitted and treated empirically for meningitis. Serial CT imaging showed resolving pneumocephalus over the course of five days. The patient's headache resolved and he did not require any other interventions. He was subsequently discharged. He was then readmitted on two more occasions for treatment of bacteremia. Completion of treatment was subsequently delayed due to additional hospital admissions. He then received one additional cycle of carboplatin and Taxol as an outpatient. Prior to being able to restart radiation, the patient was admitted again for uncontrolled pain in his shoulders and arms. MRI brain at this point showed complete resolution of the pneumocephalus. During the course of his admission, he was transferred to the medical intensive care unit due to the development of sepsis which was attributed to his immunosuppressed status secondary to chemotherapy. He also began experiencing respiratory distress. Findings on chest X-rays at that time were consistent with acute respiratory distress syndrome (ARDS) which was felt to be caused by his sepsis. No pleural effusions were noted on imaging. The patient's respiratory status continued to deteriorate despite treatment of his sepsis with antibiotics. His family declined intubation, and the patient was transferred to hospice with comfort care and expired shortly thereafter.

**Figure 5:**
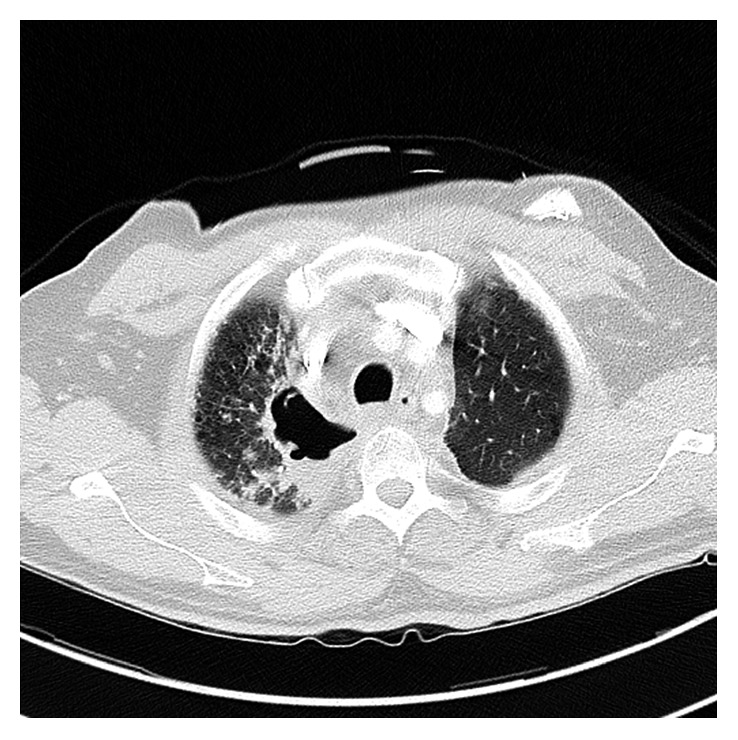
CT chest showing air-fluid levels within chest after initiation of chemoradiation.

**Figure 6:**
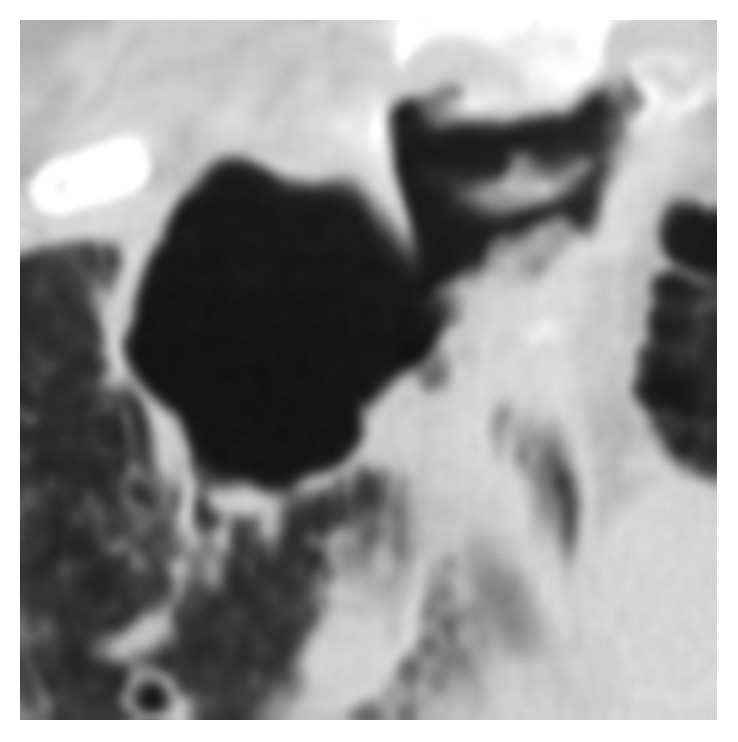
CT chest demonstrating contiguity of mass with bronchus.

In this case, the tumor invaded the C7 through T2 vertebral bodies at diagnosis. After the initiation of treatment, air was found to be present within the T1 vertebral body and extended into the right facet of T1 and T2. Additionally, air was noted in the central canal at C5-C6. Air-fluid levels within the tumor cavity were also present, indicating rapid necrosis of the mass (Figure 5). Furthermore, the air-fluid levels within the mass were contiguous with a bronchus providing an entry for air (Figure 6). Although involvement of the bronchus in a pleural-dural fistula is not described in the literature as a necessary component for the development of pneumocephalus, we believe it was a contributing factor in this patient.

## 3. Conclusions

To our knowledge, this is the first case of pneumocephalus secondary to a pleural-dural fistula described in a patient with a superior sulcus tumor who did not undergo a thoracotomy or any other operative intervention. Radiation and concurrent chemotherapy likely resulted in cell killing and necrosis and therefore may have contributed to the disruptions in the dura near the vertebral bodies in the same way that the dura could be violated in an operation. We therefore propose that the destruction of tumor cells during treatment led to the formation of a bronchopleural-dural fistula involving the lung mass, a bronchus, vertebral bodies, and central canal.

Pneumocephalus can be treated conservatively with bed rest, low head position, and antibiotic prophylaxis, and surgical repair should be considered when pneumocephalus does not resolve with conservative management [3]. Repairs have been reported using placement of a free muscle flap, fat, and omentum as well as with synthetic plugs and surgical adhesive [1, 5]. Repeat imaging of the brain of the patient in this case showed resolution of the pneumocephalus, and operative intervention was therefore deemed unnecessary. Furthermore, as in this case, patients with pneumocephalus should be treated empirically with antibiotics to prevent the development of meningitis.

In conclusion, although a potentially rare complication, pneumocephalus should be considered when patients with tumors in the superior sulcus treated with radiation then develop neurologic symptoms. Due to the devastating sequelae of pneumocephalus secondary to intracranial hypertension including seizure and stroke, prompt identification is crucial and radiation and chemotherapy should be discontinued. Operative intervention for the treatment of pneumocephalus may not be necessary in all instances if pneumocephalus resolves with conservative treatment. Supportive care, monitoring for resolution of the fistula, and antibiotics for empiric treatment of meningitis are all necessary components of care.

## Abbreviations

ISPF: iatrogenic subarachnoid pleural fistula
CT: computed tomography
MRI: magnetic resonance imaging.
